# Clinical effectiveness and cost-effectiveness of tailored intensive liaison between primary and secondary care to identify individuals at risk of a first psychotic illness (the LEGs study): a cluster-randomised controlled trial

**DOI:** 10.1016/S2215-0366(15)00157-1

**Published:** 2015-11

**Authors:** Jesus Perez, Huajie Jin, Debra A Russo, Jan Stochl, Michelle Painter, Gill Shelley, Erica Jackson, Carolyn Crane, Jonathan P Graffy, Tim J Croudace, Sarah Byford, Peter B Jones

**Affiliations:** a CAMEO Early Intervention Services, Cambridgeshire and Peterborough NHS Foundation Trust, Cambridge, UK; b Department of Psychiatry, University of Cambridge, Cambridge, UK; c Primary Care Unit, Department of Public Health and Primary Care, University of Cambridge, Cambridge, UK; d Centre for the Economics of Mental and Physical Health, King's College London, London, UK; e Department of Kinanthropology, Charles University, Prague, Czech Republic; f School of Nursing and Midwifery, Social Dimensions of Health Institute, University of Dundee, Dundee, Scotland; g National Institute of Health Research Collaboration for Leadership in Applied Health Research and Care East of England (CLAHRC-EoE), Cambridge, UK

## Abstract

**Background:**

General practitioners are usually the first health professionals to be contacted by people with early signs of psychosis. We aimed to assess whether increased liaison between primary and secondary care improves the clinical effectiveness and cost-effectiveness of detection of people with, or at high risk of developing, a first psychotic illness.

**Methods:**

Our Liaison and Education in General Practices (LEGs) study was a cluster-randomised controlled trial of primary care practices (clusters) in Cambridgeshire and Peterborough, UK. Consenting practices were randomly allocated (1:1) to a 2 year low-intensity intervention (a postal campaign, consisting of biannual guidelines to help identify and refer individuals with early signs of psychosis) or a high-intensity intervention, which additionally included a specialist mental health professional who liaised with every practice and a theory-based educational package. Practices were not masked to group allocation. Practices that did not consent to be randomly assigned comprised a practice-as-usual (PAU) group. The primary outcome was number of referrals of patients at high risk of developing psychosis to the early intervention service per practice site. New referrals were assessed clinically and stratified into those who met criteria for high risk or first-episode psychotic illness (FEP; together: psychosis true positives), and those who did not fulfil such criteria for psychosis (false positives). Referrals from PAU practices were also analysed. We assessed cost-effectiveness with decision analytic modelling in terms of the incremental cost per additional true positive identified. The trial is registered at the ISRCTN registry, number ISRCTN70185866.

**Findings:**

Between Dec 22, 2009, and Sept 7, 2010, 54 of 104 eligible practices provided consent and between Feb 16, 2010, and Feb 11, 2011, these practices were randomly allocated to interventions (28 to low intensity and 26 to high intensity); the remaining 50 practices comprised the PAU group. Two high-intensity practices were excluded from the analysis. In the 2 year intervention period, high-intensity practices referred more FEP cases than did low-intensity practices (mean 1·25 [SD 1·2] for high intensity *vs* 0·7 [0·9] for low intensity; incidence rate ratio [IRR] 1·9, 95% CI 1·05–3·4, p=0·04), although the difference was not statistically significant for individuals at high risk of psychosis (0·9 [1·0] *vs* 0·5 [1·0]; 2·2, 0·9–5·1, p=0·08). For high risk and FEP combined, high-intensity practices referred both more true-positive (2·2 [1·7] *vs* 1·1 [1·7]; 2·0, 1·1–3·6, p=0·02) and false-positive (2·3 [2·4] *vs* 0·9 [1·2]; 2·6, 1·3–5·0, p=0·005) cases. Referral patterns did not differ between low-intensity and PAU practices. Total cost per true-positive referral in the 2 year follow-up was £26 785 in high-intensity practices, £27 840 in low-intensity practices, and £30 007 in PAU practices.

**Interpretation:**

This intensive intervention to improve liaison between primary and secondary care for people with early signs of psychosis was clinically and cost effective.

**Funding:**

UK National Institute for Health Research.

## Introduction

A first episode of psychotic illness (FEP) can be devastating. Usually the illness first occurs in adolescence or early adulthood, puncturing a phase of rapid personal and social development. Some people with this disorder recover completely, but most never return to their personal developmental trajectory; others will have repeated episodes and long-term disability. Worldwide, clinical practice is increasingly predicated on early intervention, often by specialist teams in secondary care relying predominantly on patient referrals from primary care. In the past 5 years, early intervention services have come under budgetary pressures, despite strong health-economic evidence showing that prompt specialist care promotes patient recovery and is a cost-effective method.^1^ However, no evidence has yet shown that improved detection of FEP by early identification of individuals at high risk of developing psychosis might also be a cost-effective method to reduce the duration of undetected and untreated illness.

**Research in context**

**Systematic review**

We searched for studies that attempted exclusively to educate general practitioners (GPs) to recognise people at high risk of developing psychosis or those with their first episode of psychotic illness, with the aim of increasing referral of patients to specialist services. We searched PsycInfo, MEDLINE, Embase, British Nursing Index, CINAHL, HMIC, and the Social Science Citation Index using the terms “early intervention”, “psychosis” (psychotic symptoms, psychotic disorder, psychotic illness, schizophrenia), “risk” (at-risk-mental-state, prodrome, high-risk, psychotic-like), “GPs”, “primary care”, “education”, and “health services”, from Jan 1, 2001, onwards (because in this year the high risk concept was widely used and implementation of early intervention services commenced across the UK). Thesaurus and free-text terms were combined. Our search identified only two randomised controlled trials (REDIRECT^8^ and LEO CAT^7^) and two naturalistic studies.^28^, ^29^ The REDIRECT trial^8^ showed that training of GPs was insufficient to alter FEP referral rates to early intervention services, although access to specialist teams was accelerated by the intervention. By contrast, the LEO CAT trial,^7^ which combined training of GPs and patient access to a specialist service, significantly increased referral of patients with FEP to mental health services and reduced delays in treatment provision. Simon and colleagues^28^ found that increasing GPs' awareness of high-risk symptoms resulted in a significant increase in diagnostic knowledge. However, the study did not evaluate whether this resulted in more accurate or increased referrals to secondary care services. Reynolds and colleagues^29^ assessed the effect of GP training on high-risk referrals and concluded that the intervention significantly increased direct referrals to specialist teams.

**Interpretation**

Few studies, with disparate results, have attempted to educate GPs to recognise individuals at high risk of developing psychosis or those with FEP to improve patient access to secondary mental health services. None of the studies used a theory-based framework or considered the economic effects of different interventions with a randomised study design. Our cluster-randomised controlled trial shows that additional expenditure, by use of tailored intensive liaison between primary and secondary care to identify and help with the referral of individuals with early signs of psychosis, adds clinical and economic value.

General practitioners (GPs; primary care physicians) are usually the first health professionals contacted by individuals at high risk of developing psychosis.^2^ Early detection of psychosis in primary care is difficult because of the non-specific nature of its behavioural and psychological antecedents and the very low predictive value for this rare outcome.^3^ Some early intervention services in Scandinavia and Australia have developed protocols for the detection of people at high risk in primary care.^4^, ^5^ No study has assessed the clinical effectiveness and cost-effectiveness of different approaches, despite evidence that the education of GPs alone does not improve the management and identification of mental health disorders in primary care.^6^

Two previous randomised controlled trials focused on education of GPs to recognise patients with FEP.^7^, ^8^ The LEO CAT study^7^ randomly assigned an intervention that combined GP education and direct patient access to a specialist service and compared this with routine access to generic services. The intervention significantly increased the number of prompt referrals of patients with FEP to mental health services.^7^ By contrast, the REDIRECT trial^8^ showed that training of GPs alone was insufficient to alter referral rates of patients with FEP to early intervention services, although access to specialist teams was accelerated by the intervention. Neither study considered patients at high risk of developing psychosis, used a theory-based framework derived from educational research to help understand what might work to change behaviour of GPs, or assessed the economic effects of different interventions to change referral patterns.

We aimed to compare two different approaches to liaison between primary care and specialist secondary care—early intervention services for detection and early referral of young people at high risk of developing psychosis. We tested the null hypothesis that a high-intensity, theory-based, ongoing educational intervention for primary care—including liaison through named, specialist health professionals allocated to practices—is not different, in terms of clinical effectiveness and cost-effectiveness, to the provision of referral guidelines sent by post, together with ad hoc clinical contacts stemming from routine practice.

Our study is timely in view of the recent announcement from the UK Government^9^ of patient waiting time targets being extended to mental health in general, and patients with FEP in particular, and the uncertainty in financially challenged services. We investigated whether increasing the resources aimed at managing the interface from primary care to secondary care increased detection of young people at high risk of developing psychosis and early referral to a specialist early intervention team.

## Methods

### Study design and participants

Our Liaison and Education in General Practices (LEGs) study was a cluster-randomised controlled trial of primary-care general practices (clusters) in the county of Cambridgeshire and city of Peterborough (both UK). It also included an economic assessment. The protocol has been published elsewhere.^10^ Consenting primary-care practices were randomly assigned to either a high-intensity or low-intensity approach to liaison between primary care and a specialist early intervention service for psychosis (secondary care). Practices that did not consent to randomisation formed a practice-as-usual (PAU) group. Written consent was obtained from the lead GP at every practice. Our approach and methodology followed the Medical Research Council (MRC) guidelines for the design and assessment of complex interventions.^11^

104 general practices, working across 138 surgeries (some practices operated from more than one surgery with shared clinical staff), in Cambridgeshire and Peterborough were identified from the Primary Care Research Network (PCRN) East of England (now CRN Eastern Primary Care) database. Cambridgeshire and Peterborough have a total population of about 825 000 people who live in diverse socioeconomic settings—including in urban, suburban, and rural communities. In the 2011 census,^12^ 38% of this population lived in electoral wards classified with above-average levels of the English Multiple Deprivation Index.

Routine data were available for the number of high risk and FEP referrals from all practices, including those that did not consent to be randomly assigned to an intervention. These data allowed assessment of the generalisability and validity of our findings.^10^

All participating practices referred patients to an established, county-wide early intervention service for the management of FEP (CAMEO). The Cambridgeshire East Research Ethics Committee, Cambridge, UK, approved the study (reference 09/H0304/46). We only counted data for referrals of patients aged 16–35 years. We used no other exclusion criteria.

### Randomisation and masking

Practices randomly assigned to treatment groups were stratified according to three high-level factors considered a priori to be likely to be associated with referral behaviour: three geographical areas representative of the socioeconomic status in the UK (Cambridge and south Cambridgeshire [highest], Huntingdon and east Cambridgeshire, and Peterborough and Fenland [lowest]); whether GPs worked at several sites (yes *vs* no); and membership of Association of Student Practices in Cambridge (yes *vs* no), of which university students account for a high proportion (about 50%) of total list sizes.

After practices provided their consent, TJC randomly assigned practices with a computer-generated permuted sequence in blocks with 12 strata and 96 blocks, independently from the research team members who were not told of the process. This computer sequence was generated by the RALLOC command in Stata (version 11.0).^13^ Several steps were taken to keep those involved at various stages of the trial masked to the intervention groups. General practices could not be masked because the difference between the interventions was described in the information sheet required by the Cambridgeshire ethics committee. Practices randomly assigned to the high-intensity intervention would have discerned their allocation when they were contacted to arrange an educational session for the GPs. Liaison practitioners who enrolled participants and delivered the intervention could not be masked, because they had to know what intervention to deliver (eg, the high-intensity intervention). However, all patient referrals were received through a central point of contact; the administrator (part of the research team) responsible for this process was masked to the intervention allocation. All referrals were assessed by senior research clinicians who were masked to the practice allocation to an intervention. This masking process could be compromised through contact with treating clinicians but knowledge of referral origin was reduced by accommodating researchers in a different part of the building from the clinical team. Additionally, these clinicians took part in inter-rater reliability meetings once per week that were held to determine whether every referral was at high risk of developing psychosis, had FEP, or did not have psychosis. Everyone involved in this process was masked to practice origin, providing assurance that referrals from the three practice groups (high intensity, low intensity, PAU) were not being assessed differently and that raters were concordant. The trial statistician (JS) was not masked to practice allocation, but analysed only the count data provided.

### Procedures

Practices were provided consent to participate between Dec 22, 2009, and Sept 7, 2010. Referral activity by primary-care practices and the results of specialist clinical assessments were recorded for 2 years after random allocation to an intervention group between Feb 16, 2010, and Feb 11, 2011.

The Comprehensive Assessment of At-Risk Mental States (CAARMS) interviews, semistructured and designed to detect prodromal symptoms of psychotic disorders to suggest which patients are at high risk of transition to FEP, were done by senior research clinicians trained by experts involved in previous trials that used it, such as the MRC EDIE trial.^14^ CAARMS is also used to determine whether an individual meets criteria for high risk or FEP. It is divided into four main symptom domains: unusual thought content, non-bizarre ideas, perceptual abnormalities, and disorganised speech. This interview system's scores include intensity and frequency of these symptoms, and has good-to-excellent concurrent, discriminatory, and predictive validity in this setting and excellent inter-rater reliability.^15^ Inter-rater reliability was based on 104 evaluations by three independent raters and showed an excellent overall agreement for all four CAARMS domain scores (intra-class correlation mean 0·98; SD 0·1; range 0·96–1).

The main element of the low-intensity intervention was a postal information campaign, comprising a specifically designed laminated leaflet (appendix). The leaflet provided guidelines to help GPs identify and refer individuals at high risk or those with FEP. It was posted to the practices in the low-intensity group every 6 months during the study. The leaflets were integrated within the high-intensity educational programme (high-intensity intervention) and distributed at the same frequency as low intensity to compare the two groups.^10^

The high-intensity intervention comprised a tailored education and liaison approach between primary and secondary care, designed using the principles of the MRC framework for the development and evaluation of complex interventions^11^ and evidence about effective educational interventions in primary mental health care.^16^ We addressed the absence of an explicit theoretical framework in the design of many educational interventions to change professional practice^17^ by using the Theory of Planned Behaviour (TPB),^18^, ^19^ which predicts intentions and behaviour in relation to clinical practice.^20^ This theory proposes that the identification of individuals at high risk of developing psychosis in primary care is predicted by the strength of a GP's intention to identify these individuals. This intention is affected by three predictor variables: whether the GP is in favour of identification (attitude); the intensity of social pressure the GP perceives to identify early psychosis (subjective norm); and how much the GP feels in control of this identification process (perceived behavioural control).^19^

Use of the TPB to design interventions requires the development of a questionnaire to allow the identification and measurement of specific beliefs associated with each construct (ie, intention, attitude, subjective norm, and perceived behavioural control). In accordance with the TPB guidelines,^21^, ^22^ pilot work was undertaken before this study to identify accessible behavioural, normative, and control beliefs. This work generated a questionnaire that was used to measure factors that affected a GP's identification of individuals at high risk of developing psychosis. The pilot work also guided the development of the materials and strategies included in the intervention, which were aimed at encouraging GPs to identify individuals at high risk by incorporating apposite knowledge and skills into their practice.^23^

These techniques were delivered and facilitated by three liaison practitioners over the 2 year intervention period. All three practitioners were experienced mental health professionals responsible for delivering the intervention to the consenting practices within one of the three chosen geographical areas in Cambridgeshire. The main behavioural change technique consisted of two practice-based educational sessions. An initial 1 h educational session was on detection of high-risk individuals for practices when they started the trial and was followed 1 year later by a booster 1 h session to reiterate the main messages, consolidate skills and knowledge, discuss particular practical scenarios that emerged during the course of the study, and to adjust or improve ongoing intensive liaison techniques if needed. This approach allowed the intervention to be tailored to meet the specific needs of every practice. Together with other components of the intervention, this allowed comparisons of cost-effectiveness between the resource intensive strategy (high-intensity intervention) and simple postal information campaign (low-intensity intervention).^10^

Practices that did not consent to be randomly assigned between the two interventions continued to receive postal leaflet information about early signs of psychosis, but without a specific focus on patients at high risk and did not receive the leaflet as often as the low-intensity campaign (PAU: once per year *vs* low-intensity and high-intensity: twice per year).

### Outcomes

The primary outcome was count data (ie, number) of high-risk referrals to the early intervention service analysed per practice (the yield) during the 2 years of this study. New patient referrals during the trial who were clinically assessed by the study team were stratified into those who met criteria for high risk or FEP according to CAARMS^15^ (psychosis true positives) and those who did not fulfil the criteria (false positives). Additionally, the economic evaluation assessed the cost-effectiveness of both interventions in terms of detection of true-positive patients (at high risk or with FEP).

### Statistical analysis

We used sample size formulae for Poisson outcomes in a cluster-randomised controlled trial design, comparing high-intensity and low-intensity interventions, and an assumption that the high-intensity intervention would double the number of referrals of patients at high risk of developing psychosis to secondary care compared with the low-intensity intervention. For power of 80% with a significance level at 0·05 (two-sided), referral counts expressed as an incidence rate of referrals in the low-intensity group of 40 per 100 000 person-years,^24^ an anticipated incidence rate in the high-intensity group of 80 per 100 000 person-years, 2000 person-years per site (average surgery list size for patients aged 16–35 years per 2 years of study), and a coefficient of variation estimated at 0·15, our calculations showed we needed a sample size of 31 surgeries (sites) in each arm.

The main outcome was count data, the yield, so our primary statistical approach was Poisson regression. If the assumptions of Poisson regression were not met (eg, over-dispersion), we used alternative models such as quasi-Poisson, Poisson with robust standard errors, or negative binomial regression models. If excessive numbers of zeros were noted, we then used zero inflated models and hurdle models. The fit of the model to the data was assessed by comparison of model log-likelihoods (between Poisson and negative binomial model) or the Vuong test^25^ (between Poisson and zero inflated model). Subsequently, the best fitting model was selected, although the overall pattern of results showed no difference between models. Analysis was by modified intention to treat. All practices were considered to remain in their allocated groups irrespective of subsequent engagement in the trial interventions and other matters, unless practices closed, withdrew, or became ineligible from the study immediately after randomisation.

Results were adjusted for surgery size, regarding the number of GPs working in each site as an offset variable in the model. As our main predictors (low intensity, high intensity, or PAU) were categorical variables, we first set the high-intensity group as the reference. However, this choice did not allow for direct comparisons between the low-intensity and PAU groups so, in this case, we then used the low-intensity group as the reference. We also used *F* tests, Kruskal-Wallis χ^2^ test, Pearson's χ^2^ test, and Fisher's exact test to compare demographic characteristics of the general practices. For inter-rater reliability of CAARMS, we used intraclass correlation coefficient.^26^ A sensitivity analysis assessed the effect of some individuals refusing assessment after referral. All analyses were done using the statistical package R version 3.1.2.^27^

### Economic analysis

The economic evaluation aimed to explore the cost-effectiveness of the high-intensity and low-intensity interventions compared with the PAU group, using decision analytical modelling. We constructed a decision tree in Excel 2013 to model the care pathways of the young people in the trial and to assess the costs and effects in 2 years associated with the two active interventions and PAU. Costs chosen in the analysis were those relevant to the UK's National Health Service (NHS) and social care in England and included costs of the high-intensity and low-intensity interventions, diagnosis of referrals who did not meet criteria for high risk or FEP (false positives), diagnosis and treatment of patients identified as high risk and FEP (true positives), and the subsequent treatment for high risk and FEP who were not identified (false negatives). The cost of true-negative cases was assumed to be zero.

Cost-effectiveness was expressed as the incremental cost per additional true-positive case (high risk or FEP) identified. Input data were obtained mainly from this cluster-randomised controlled trial, with economic data gathered from a service use schedule designed for use with an early intervention sample. This schedule was completed by the individuals at high risk or with FEP who were referred to CAMEO and repeated at 6, 12, 18, and 24 months. Data for input parameters not available from the trial—eg, for patients at high risk or with FEP not identified in the study (false negatives)—were estimated using published data (appendix). Full details about the economic methods are provided in the appendix.

### Role of the funding source

The funder of the study had no role in study design, data collection, data analysis, data interpretation, writing of the report, or in the decision to submit for publication. JP, HJ, DAR, JS, SB, and PBJ had full access to the raw data in the study. The corresponding author had final responsibility for the decision to submit for publication.

## Results

**Figure 1:**
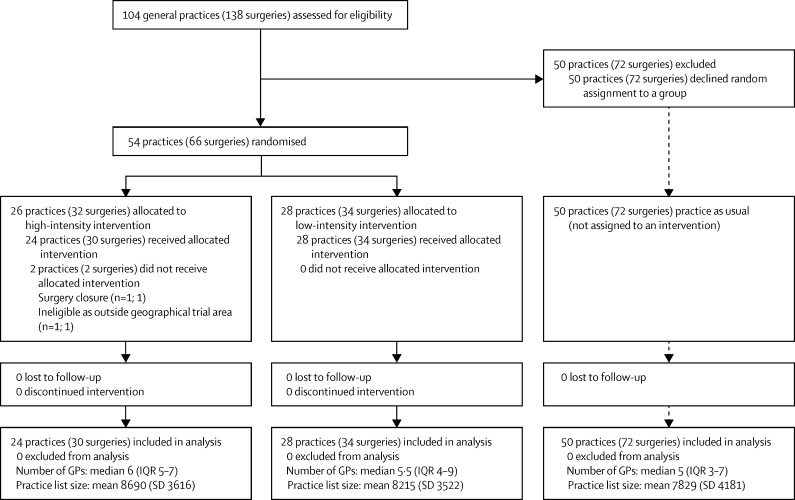
Trial profile GPs=general practitioners.

Of the 104 general practices (138 surgeries) in Cambridgeshire and Peterborough eligible to participate, 54 practices (66 surgeries) consented to be randomly assigned between Dec 22, 2009, and Sept 7, 2010. 28 practices (34 surgeries) were assigned to the low-intensity group and 26 practices (32 surgeries) to the high-intensity group (figure 1). In the high-intensity group, two practices (two surgeries) were excluded because one practice closed soon after consenting and its patients dispersed to other practices in the study, and the other was incorrectly on the list of eligible practices because it was outside the county and catchment area of the early intervention service. 50 practices (72 surgeries) did not consent to randomisation and thus formed the PAU group (figure 1). 34 (68%) of these practices provided no reason for not consenting, 14 (28%) attributed their decision to high workload, and two (4%) to a large number of ongoing research projects. No PAU practice had a specific alternative approach for liaison with secondary care.

**Figure 2:**
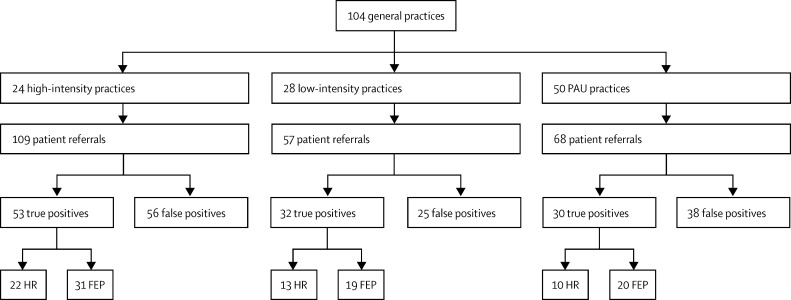
Number and type of referrals by general practices in Cambridgeshire and Peterborough PAU=practice as usual. HR=high risk of developing psychosis. FEP=first-episode of psychotic illness.

Table 1 shows baseline characteristics of practices in the high-intensity, low-intensity, and PAU groups; we did not note any significant differences between groups. During the 2 year intervention period, 234 patient referrals were made to the specialist early intervention in psychosis service (CAMEO) from the study practices for assessment of possible psychotic symptom (figure 2). The mean number of referrals during the 2 years from the high-intensity group was 4·5 per practice (SD 3·1), 2·0 (2·55) from the low-intensity group, and 1·4 (1·5) from the PAU group.

39 (17%) referrals received during the 2-year intervention were not included in the analysis because the individuals declined clinical assessment; therefore, their clinical status could not be ascertained. 16 (41%) patients were referred by high-intensity practices, seven (18%) patients by low-intensity practices, and 16 (41%) patient referrals were made by practices in the PAU group.

**Figure 3:**
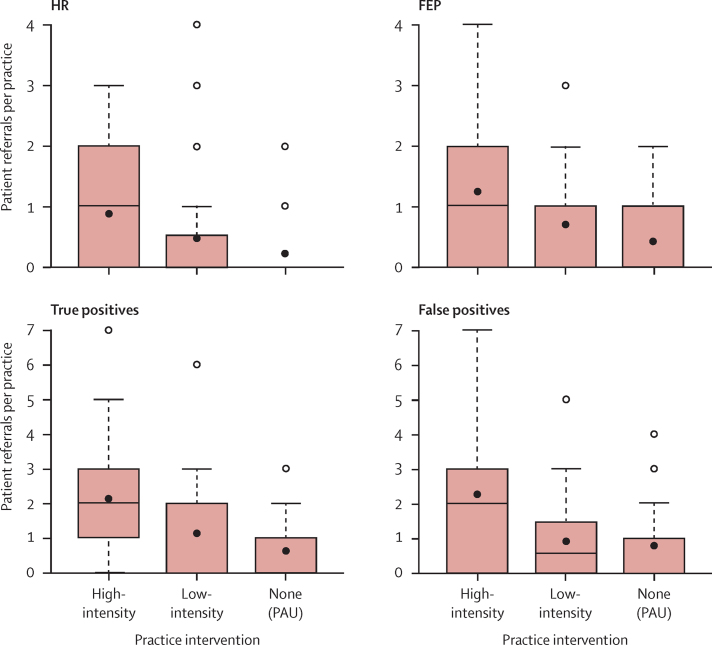
Box plot of the distribution of patient referrals from primary care to secondary care HR=high risk of developing psychosis. FEP=first episode of psychotic illness. PAU=practice as usual. Lower bounds show the 25th percentile. Upper bounds show the 75th percentile. Line represents the median. Black dot represents the mean. White dots show the outliers.

In terms of mean numbers of referrals per practice (figure 3), high-intensity practices referred more people who were subsequently identified to be at high risk (0·9 [SD 1·0]) or with FEP (1·25 [1·2]; combined as psychosis true positives) than did low-intensity practices (high risk 0·5 [1·0]; FEP 0·7 [0·9]) and PAU general practices (high risk 0·2 [1·5]; FEP 0·4 [0·6]). The high-intensity practices referred the most true-positive cases (patients at high risk or who had FEP; 2·2 [1·7]; low intensity 1·1 [1·7]; PAU 0·6 [0·85]). The same pattern was noted for referrals of false positives (patients not at high risk or who did not have FEP; high-intensity practices 2·3 [SD 2·4], low-intensity practices 0·9 [1·2], and PAU 0·8 [1·1]). However, 81 (68%) of individuals without psychosis (false positive) who were diagnosed in this trial were directed to other mental-health-related services for help with their problems; 23 (28%) needed input from secondary or tertiary mental health services. 58 (72%) individuals were referred to Improved Access to Psychological Therapies (IAPT) services in primary care, wherein they would receive up to 20 sessions of largely cognitive behavioural therapies.

The best fitting model for every group of referrals was reported (table 2). High-intensity practices referred more FEP (incidence rate ratio [IRR] 1·9, 95% CI 1·05–3·4, p=0·04) and true-positive cases (2·0, 1·1–3·6, p=0·02) than did the low-intensity and PAU practices (table 2). High-intensity practices also referred the most false-positive cases (2·6, 1·3–5·0, p=0·005). The low-intensity postal campaign seemed to have very little effect on number of referrals compared with the PAU group. The number of referrals from high-intensity practices was higher than from PAU in all monitored referral groups (figure 2). Sensitivity analyses, including the 39 patients who declined assessment, did not modify any of these results (further details about these analyses and the statistical model-building that led to these results are available from the authors).

Total costs and effect on the number of referrals per practice during the 2 year follow-up were compared between intervention groups (table 3). Compared with both the low-intensity intervention and PAU group, the high-intensity intervention was more effective at identifying patients at high risk of developing psychosis or with FEP and was associated with lower total costs per practice, mainly as a result of fewer false-negative cases (patients at high risk and FEP not identified, but who are assumed to be associated with later treatment costs; appendix). Thus, the high-intensity intervention was more cost effective than both the alternative liaison approaches. These results were robust to one-way and probabilistic sensitivity analyses (appendix; patient level data are available from the authors on request).

## Discussion

Our cluster-randomised controlled trial showed that tailored and intensive liaison between primary and secondary care to detect people with early signs of psychosis and to help improve their access to mental health services can be clinically and cost effective. Our theory-based, high-intensity intervention was more effective than a postal information campaign at increasing number of referrals to specialist care for patients with FEP or at high risk of developing psychosis (panel). This intervention was costly both in terms of resources and time. However, the economic decision model suggests that this additional expenditure has the potential to generate subsequent savings through earlier detection and referral to specialist early intervention services.

Our work was informed by the LEO CAT study,^7^ which assessed the effectiveness of educating GPs and provision of a specialist service to help with the identification of a FEP. However, important differences exist between our study and the LEO CAT study. First, we lowered the threshold for psychotic symptoms and attempted to educate GPs to also identify individuals at high risk. Second, our overall sample was larger and the trial covered a more diverse socioeconomic area (including urban, suburban, and rural settings) and an established early intervention service for psychosis. Third, we focused on the educational package. In the LEO CAT trial,^7^ the intervention group also had direct access to the LEO CAT clinical team designed to work closely with GPs, whereas the control group received standard care provided by community mental health services. Therefore, it is not possible to determine if one or both of these elements resulted in the increased number of referrals in the LEO CAT study. Fourth, our educational intervention was developed using a theory-based framework derived from educational research. Despite the success of the intervention in the LEO CAT study,^7^ it is difficult to identify which specific factors changed the referral behaviour in GPs. The absence of a theoretical framework underpinning interventions used in previous studies has obscured understanding of the behavioural determinants (what to target) and the selection of techniques to change these determinants (how to target them). As a result, such interventions are difficult to replicate, which precludes their development across different contexts and populations.^30^ Finally, we included an economic analysis; if the intervention were to prove costly in terms of resources and time, the benefit of any number of increased referrals might be negated.

Our intervention doubled the number of referrals of patients at high risk, matching our prediction, but the confidence limit for this effect included unity, failing to reject the primary null-hypothesis. However, this effect was matched by almost twice the number of referrals of patients with FEP and false-positive cases so we believe it is likely to be true. Growing evidence suggests that psychosis represents a continuum, with psychosis proneness and mild psychotic symptoms at one end and schizophrenia and other psychotic disorders at the other.^31^ Individuals in high-risk states and those with FEP in our trial sought help, and thus probably together represented the severe end of this psychosis continuum rather than different categorical entities from each other.^32^ Accordingly, we grouped individuals at high risk and those with FEP as psychosis true positives, showing the overall number of individuals seeking help. Their combined referral numbers were doubled by the high-intensity intervention. We believe that our high-intensity intervention enhanced the detection of individuals with psychotic symptoms in primary care and their referral to the early intervention service.

In our study, high-intensity practices also referred more people without psychosis (false positives) than did the low-intensity intervention or PAU practices. A possibility for this might be that the high-intensity intervention raised awareness and increased sensitivity in GPs' referral behaviour in general, but had poor specificity to correctly identify individuals at high risk. Most patients identified as false positives had substantial impairment in their mental health, involving, in some instances, psychotic-like experiences that did not reach the CAARMS threshold criteria for high risk; from the GPs' point of view the referrals were correct. These patients needed treatment and were referred to IAPT or secondary or tertiary mental health services. We considered the cost of diagnosing these referrals, but did not collect economic data associated with the treatment that they subsequently received elsewhere in the NHS. This information will be useful in future economic assessments in similar settings.

Another important finding is that the leaflet posted to GPs (low intensity) was no more effective in generating referrals of individuals with FEP or at high risk of developing psychosis than PAU (no intervention). This result has implications for future postal campaigns and referral guidelines to raise awareness of psychotic symptoms; although a relatively inexpensive strategy, our findings suggest that it has little or no worth.

Every practice randomly assigned to the high-intensity intervention was offered support and training in the form and frequency that best suited their particular needs, on the basis of the information gathered from the TPB sessions. During the 2 years of the intervention the liaison practitioners were rarely called upon by participating GPs for advice and support regarding potential referrals of individuals at high risk or to request additional training in between the two prearranged educational sessions. High GP workload and scarce time might have contributed to GPs not requesting additional assistance,^33^, ^34^ although GPs were willing to engage in discussions about previous referrals during the second educational session. Accordingly, liaison practitioners might have covered more practices, thus increasing cost-effectiveness.

Our study has several limitations. Our original intention was to randomise all practices in the study area without consent and so exceed the numbers of clusters needed in each arm indicated by our power calculations, but the research ethics committee decided that this approach was not acceptable.^10^ Furthermore, our assumptions about the general epidemiology of psychosis have been updated since the protocol was developed. Areas of low income have a high prevalence of psychosis^35^ and practices in our most deprived areas were most likely to decline being randomly assigned to an intervention, therefore we might have missed yield. This problem was not helped by initially including two ineligible practices. Thus, our statistical power was lower than intended, which could account for why the confidence limits for our primary effect included unity whereas those for FEP and the combined psychosis true-positive outcome were narrower—about the same doubling in the number of referrals.

The random assignment process was concealed and we took steps to mask allocation along with other design features to restrict the likelihood that bias led to our results. These approaches were robust within the limits of pragmatism, but probably not perfect. Verification of masking of the central administrator was shown by the fact that no referrals were rejected. Not all the surgeries had a comparable overall educational experience, GP staff changed during the trial, and the intention-to-treat approach was conservative. We militated against bias by offering practices several visits to ensure the maximum number of clinicians attended each session. However, some GPs inevitably arrived late or left early due to clinical commitments. Nevertheless, such GPs could still have been influenced by the cluster-level intervention, as ascertained by the authors of the TPB guidelines.^21^ These nuances were not measured in our trial because the TPB questionnaires were anonymous to increase the chance of authentic responses. The time that the effect of the intervention persists and the optimum number of refresher sessions that are needed are not known. Future research should investigate these factors to achieve a balance between intervention effectiveness and cost-effectiveness, as additional educational booster sessions could be either unproductive or crucial to sustain identification of individuals at high risk or with FEP.

For more about **IAPT** see http://www.iapt.nhs.uk

## Figures and Tables

**Table 1:** Baseline characteristics of high-intensity, low-intensity, and practice-as-usual general practices

		**High intensity (n=24)**	**Low intensity (n=28)**	**Practice as usual (n=50)**	**p value**
Number of GPs		6 (5–7)	5·5 (4–9)	5 (3–7)	NA
Practice patient list size		8690 (3616)	8215 (3522)	7829 (4181)	0·66*; 0·17†‡
Number of additional sites		0 (0–0)	0 (0–0)	0 (0–1)	NA
University affiliated surgery					0·09§; 0·06¶; 0·07†§; 0·06†¶
	Yes	3 (13%)	4 (14%)	1 (2%)	
	No	21 (88%)	24 (86%)	49 (98%)	
Number of GPs working across several sites					0·25§; 0·29¶; 0·15†§; 0·13†¶
	Yes	6 (25%)	6 (21%)	19 (38%)	
	No	18 (75%)	22 (79%)	31 (62%)	
Practices per region					0·58§; 0·57¶; 0·26†§; 0·27†¶
	Huntingdon and east Cambridgeshire	8 (33%)	10 (36%)	12 (24%)	
	Peterborough and Fenland	7 (29%)	9 (32%)	23 (46%)	
	South Cambridgeshire	9 (38%)	9 (32%)	15 (30%)	

Data are median (IQR), mean (SD), or n (%), unless otherwise stated. GPs=general practitioners.

* *F* test.

† Statistical differences between practices that consented (high intensity plus low intensity) and did not consent (practice as usual) to be randomly assigned.

‡ Kruskal-Wallis χ^2^ test.

§ Pearson's χ^2^ test.

¶ Fisher's exact test.

**Table 2:** Comparison of effectiveness (incidence rate ratios) between high-intensity, low-intensity, and practice-as-usual (PAU) general practices

	High risk of developing psychosis*	**First episode of psychotic illness**†	True positives*	False positives*
**Reference: low intensity**				
Intercept	0·1 (0·03–0·2); p<0·0001	0·1 (0·07–0·2); p <0·0001	0·2 (0·1–0·3); p <0·0001	0·1 (0·09–0·2); p <0·0001
High *vs* low	2·2 (0·9–5·1); p=0·08	1·9 (1·05–3·4); p=0·04	2·0 (1·1–3·5); p=0·02	2·6 (1·3–5·0); p=0·005
PAU *vs* low	0·5 (0·2–1·5); p=0·2	0·7 (0·4–1·3); p=0·2	0·6 (0·3–1·2); p=0·1	1·0 (0·5–1·9); p=1·0
**Reference: high intensity**				
Intercept	0·2 (0·1–0·2); p<0·0001	0·2 (0·1–0·3); p<0·0001	0·4 (0·3–0·5); p<0·0001	0·4 (0·3–0·6); p<0·0001
Low *vs* high	0·5 (0·2–1·1); p=0·08	0·5 (0·3–0·9); p=0·035	0·5 (0·3–0·9); p=0·017	0·4 (0·2–0·8); p=0·005
PAU *vs* high	0·3 (0·1–0·6); p<0·0001	0·4 (0·2–0·6); p<0·0001	0·3 (0·2–0·5); p<0·0001	0·4 (0·2–0·7); p<0·0001

Data are incidence rate ratios (95% CI), unless otherwise stated.

* Negative binomial test.

† Poisson test.

**Table 3:** 2-year costs and cases identified per general practice, by intervention group

	**Mean number of true-positive cases identified per practice (SD)**	**Total 2-year cost per practice**
High intensity	2·2 (1·7)	£26 785
Low intensity	1·1 (1·7)	£27 840
Practice as usual	0·6 (0·85)	£30 007
