# Supplementary material for: Clinical effectiveness and cost-effectiveness of tailored intensive liaison between primary and secondary care to identify individuals at risk of a first psychotic illness (the LEGs study): a cluster-randomised controlled trial
Source: Lancet Psychiatry. 2015 Nov;2(11):984–93. doi: 10.1016/S2215-0366(15)00157-1 (PMC4641188; doi:10.1016/S2215-0366(15)00157-1)
Supplement: Supplementary appendix [file mmc1.pdf]

THE LANCET Psychiatry

Supplementary appendix

This appendix formed part of the original submission and has been peer reviewed. We post it as supplied by the authors.

Supplement to: Perez J, Jin H, Russo DA, et al. Clinical effectiveness and cost-effectiveness of tailored intensive liaison between primary and secondary care to identify individuals at risk of a first psychotic illness (the LEGs study): a cluster-randomised controlled trial. *Lancet Psychiatry* 2015; published online August 19. http://dx.doi.org/10.1016/S2215-0366(15)00157-1.

Economic evaluation methods

Aim

The economic evaluation aimed to explore the cost-effectiveness of the high and low intensity interventions in comparison to practice as usual for the identification and referral of young people at high risk of psychosis (HR) or meeting criteria for first episode psychosis (FEP), where cost-effectiveness was expressed as the incremental cost per additional true positive (HR or FEP) identified.

Design

The economic evaluation employed a decision modelling design in order to incorporate all possible categories of young people in this screening study: true positive, false positive, true negative and false negative. The trial only collected data on the first two groups, those identified and referred to the specialist early intervention service, and was not set up to identify or collect data on true or false negatives. As a result, reporting of within-trial cost-effectiveness was considered inappropriate, since it would fail to include all important costs and effects of the interventions under evaluation. Decision modelling allows these hidden, but important, costs to be incorporated, providing more meaningful assessment of the full economic impacts of each intervention.

True negatives were excluded from the economic evaluation as they were assumed to have zero cost. However, false negatives must be included as they are HR or FEPs who, although not initially identified by participating general practices, may go on to incur health service costs and whose outcomes may deteriorate as a result of late identification. False positives, although not HR or FEP, are included as they still incur costs (and potentially outcome

changes) as a result of referral to the specialist early intervention service, which as a minimum will undertake an initial assessment of risk status.

Model structure

A decision tree was constructed in Excel 2013 to model the care pathways of the young people in the trial and assess the costs and effects over two years associated with the two active interventions and practice as usual.

The model structure was identical for all three intervention groups and is shown in Figure 1. The model assumes that for each intervention group, there are four types of patients: HR identified, HR missing, FEP identified and FEP missing. HR identified and HR missing may either make a transition to psychosis or not make such transition. It is assumed all transitions will happen at the end of year 1. All input parameters and the source of the data are reported in Table 1. Input data were obtained primarily from the trial, with published data used where necessary. Due to the short time horizon of this economic analysis (two years), costs and health outcomes were not discounted.

Model parameters

Number of identified and missing HR and FEP

The number of identified HR and FEP was taken from the trial data. The number missing, however, is unknown. In the absence of any robust data on the true incidence of HR and FEP in Cambridgeshire and Peterborough, it was assumed that the true incidence is equivalent to the highest rates identified in the trial (the high intervention group) and thus the missing rates for the low intervention group and the practice as usual group is calculated as the difference

between the rate identified in the high intervention service and the service of interest. Resulting rates of identified and missing HR and FEP are reported in Table 1.

Model probabilities

The transition rate from identified HR to FEP in the randomised trial was calculated to be 0.12 (12.0%) over the two year follow-up. Transition rates from unidentified HR to FEP were not collected by this trial, and were therefore taken from published studies. Three naturalistic studies reporting 12-month transition rates from unidentified HR to FEP were identified, with rates of 35.0%[1], 40.80%[2] and 54.0%[3]. For this analysis, we took the most conservative estimate (biased against interventions that are more successful at identification) and assumed that the transition rate to psychosis for unidentified HR was the lowest rate of 35.0%.

Model costs

Costs considered in the analysis were those relevant to the National Health Service in England and included the cost of the high and low intensity interventions, the cost of diagnosing referrals who did not meet criteria for HR or FEP (false positives), the cost of diagnosing and treating identified HR and FEP (true positives) and the cost of treatment for HR and FEP who were not identified (false negatives). The cost of true negatives was assumed to be zero. Costs by intervention are summarised in Table 1.

Intervention costs: Intervention costs for the practice as usual group were assumed to be zero. The cost of the low and high intensity interventions were estimated on the basis of information provided by the study team on the nature and composition of the interventions, including printing and postage for information leaflets, newsletters and information packs,

DVD costs, and liaison practitioner and general practitioner time devoted to the education sessions. Each intervention component and their respective costs are reported in Table 2.

Cost of diagnosis: The cost of diagnosing a false positive during the course of an initial assessment session with the specialist early intervention service was assumed to be equivalent to the weighted average cost of all adult outpatient attendances for mental health of £100.[4]

Treatment costs for identified HR: The costs of HR participants were estimated using service use data collected in the trial. Data were collected using a version of the Adult Service Use Schedule (AD-SUS), a measure of service use developed and applied in various mental health populations, which was adapted for application to an early intervention sample (available from the authors). The Early Intervention AD-SUS was completed by trial participants in interview at 6, 12, 18 and 24-months after study entry. The measure covered use of all health and social care services over the previous six months, as well as socio-demographic information, employment status and pathways to care. Criminal activity was also recorded, but only one HR young person reported having committed any crimes, resulting in substantial variance. Given the small sample sizes involved, these costs were excluded from the current analysis to avoid undue influence of this one outlier, which may be the result of chance. Productivity losses as a result of days off work due to illness were considered, but few of the young people in the trial were working and of these, very few reported any days off work. Productivity losses were therefore negligible. Costs were calculated by applying relevant 2012 unit costs from published sources.[4, 5]

Treatment costs for unidentified HR: Data to estimate the cost of unidentified HR were taken from a decision analytic model which compared the cost-effectiveness of an early

identification and treatment service with care as usual, for people with an at-risk mental state for psychosis.[6] The authors assumed that in the absence of an early identification service for HR, an individual experiencing prodromal symptoms would have six contacts with a counsellor and six contacts with a GP during the first 12 months and they further assumed that this rate of contact with GPs and counsellors would be halved during the second 12 months. In the absence of alternative evidence, these assumptions were used to estimate the treatment cost for missing HR at £732 for Year 1 and £366 for Year 2.

Treatment costs for identified and unidentified FEP: Data were available to estimate the cost of treating FEP from the Lambeth Early Onset (LEO) trial,[7] comparing the costs and cost-effectiveness of an early intervention service in London with standard care, for individuals in their first episode of psychosis. Since early intervention services have now become standard practice in the study sites, and many other parts of the country, the cost of the early intervention service group were used to estimate the cost of FEP in the current study. Evidence suggests that longer duration of untreated psychosis (DUP) is associated with increased treatment costs.[8] Data from the LEO study allowed separation of the costs of those classified as having long DUP (>8 weeks) or short DUP (≤8 weeks). For the current study, we assumed that those FEP identified and referred by participating general practitioners included a mixture of both long and short DUP, so the average cost of the full LEO sample was applied (£8,945 per annum). For unidentified FEP, a long DUP was assumed, which was calculated as the average of the sub-sample of those with long DUP in the LEO study (£10,351 per annum).

Treatment costs for transition from HR to FEP: For identified and unidentified individuals initially assessed as HR who transitioned to FEP during the two-year follow-up (transition

assumed to take place at the end of year 1 for the purpose of the model), costs in year 2 were assumed to be the same as for identified FEP (£8,945) and unidentified FEP (£10,351), respectively.

Sensitivity analysis

The following key assumptions were considered for their likely robustness and for variation in sensitivity analyses:

1. The number of missing HR and FEP: In the absence of any robust data on the true incidence of HR and FEP in Cambridgeshire and Peterborough, it was assumed that the true incidence is equivalent to the highest rates identified in the trial (the high intervention group). Although this may potentially underestimate the true incidence, the absolute difference between groups remains the same irrespective of the rate chosen, thus sensitivity analysis was not considered necessary for this comparative analysis.
2. Transition rates from unidentified HR to FEP: We took the most conservative estimate from available evidence in the literature – the lowest rate – which would bias against interventions that are more successful at identification and thus would work against the high intensity intervention, and to a lesser extent the low intensity intervention, compared to practice as usual. Testing the alternative rates available would not therefore change the conclusion of the economic evaluation.
3. Treatment costs for identified HR: Treatment costs for identified HR (reported in Table 1) were found to be substantially higher for PAU participants than the low or high intervention participants, perhaps suggesting the high and low intensity interventions have an impact not just on the number of cases identified, but also on how early they are identified. However, to guard against the possibility that these

differences are due to chance, rather than a true difference, the cost of identified HR for all three groups was replaced with the mean of the low and high intensity group costs (£1046), thus removing any bias against PAU that may be due to chance.

4. Treatment costs for unidentified HR: Data to estimate the cost of unidentified HR were taken from assumptions made in an existing decision analytic model.[6] In the absence of alternative current evidence, it was not possible to hypothesise how reliable these estimates might be. However, the treatment costs applied were low in comparison to the cost of identified HR recorded in the current study, so this assumption was considered to be conservative.
5. Treatment costs for identified and unidentified FEP: We assumed that those FEP identified and referred by participating general practitioners included a mixture of both long and short DUP, which was assessed by the clinical team as a conservative assumption. For unidentified FEP, a long DUP was assumed. This figure was considered by the clinical team to be realistic but not conservative so deterministic one-way sensitivity analysis was performed varying the percentage with long DUP from 100%, applied in the main analysis, to 80% long DUP which was considered to be a conservative estimate, in order to assess the robustness of the results to this assumption.

Uncertainty was explored using probabilistic sensitivity analysis, a form of analysis which involves assigning probability distributions to model parameters (costs and effects) and sampling at random from the distributions, in this case 5,000 times, to generate an empirical distribution for each parameter.[9] To represent uncertainty in costs, a gamma distribution was fitted to reflect the fact that cost data are commonly skewed in nature. For effects, we initially assigned a normal distribution, but this generated a number of negative values for

effectiveness data, so a gamma distribution was used instead. All parameters for the probabilistic sensitivity analysis are recorded in Table 3.

Cost-effectiveness planes are presented which illustrate differences in costs and effects between different strategies using scatter points generated by the probabilistic sensitivity analysis that represent multiple cost and effectiveness pairs. Cost-effectiveness planes consist of four quadrants, where the x-axis represents the additional level of effectiveness generated by one intervention compared to another (in this case the high intensity intervention compared to either the low intensity intervention or PAU) and the y-axis represents the additional cost of one intervention compared to another. Cost-effectiveness acceptability curves are also presented, which represent the probability of one intervention being more cost-effective than the alternatives as a function of the willingness to pay for a unit improvement in outcome.[10] Since willingness to pay for an improvement in true positive cases identified is not known, a range of possible values of willingness to pay are plotted.

Protocol changes

1. Economic perspective: Within the chosen perspective, data on productivity losses and criminal activity were collected in the trial, but were excluded from the analysis due to the very limited relevance of these resources, as described above.

2. Within-trial evaluation: Although a within-trial evaluation had been planned, the lack of data on the population of false positives (those HR or FEP that practices fail to identify but who are still likely to be receiving services and incurring costs) meant the results were misleading and thus a decision model, allowing these to be included, was selected.

3. Long-term extrapolation: Longer-term extrapolation was explored, but review of the literature did not locate appropriate data (on longevity of the intervention or longer term outcomes for HR and FEP individuals), so a longer-term model was considered premature.

Results

Two-year costs and effects per general practice by intervention group (reported in the main manuscript) are summarised in Figure 2. Compared to both the low intensity intervention and PAU, the high intensity intervention was found to be more effective at identifying HR and FEP and associated with lower total costs per practice, primarily as a result of fewer false negatives (HR and FEP that are not identified but are assumed to be associated with treatment costs at a later point). Thus the high intensity intervention dominates both the alternative interventions in terms of cost-effectiveness.

One-way sensitivity analysis did not change these findings. Varying the cost of identified HR patients to remove the bias against PAU reduced the total cost of the PAU group per practice (from £30,007 to £29,367), but the reduction was not enough to change the ranking between the groups (low intensity £27,931 per practice; high intensity £26,603 per practice), thus the high intensity intervention remained dominant. Similarly, when the percentage of unidentified FEP with long DUP was reduced from 100% to 80%, the cost advantage for the high intensity group remained, although by a reduced amount (£165 cheaper than the low intensity group per practice; £1,913 cheaper than PAU).

The results of the probabilistic sensitivity analysis are illustrated in Figures 3-6. Figures 3, 4 and 5 show the cost-effectiveness planes for low intensity versus PAU, high intensity versus

PAU, and high intensity versus low intensity, respectively. In all three figures, the scatters of cost and effectiveness pairs fall mainly to the right of the y-axis, reflecting the greater level of effectiveness seen for both low and high intensity compared to PAU and for high intensity compared to low intensity. Points are more evenly spread above and below the x-axis, reflecting similar levels of total cost across the three groups. In all three comparisons, the majority of the scatter points fall into the Southeast quadrant (representing the situation where the intervention under consideration is more effective and less effective, and thus dominates, the comparison intervention) and the Northeast quadrant (representing the situation where the intervention under consideration is more effective but also more costly than the comparison intervention). For each comparison, the proportion of replications falling into each quadrant is reported in Table 4.

The cost-effectiveness acceptability curve, presented in Figure 6, continues to support the dominance of the high intensity intervention compared to either the low intensity intervention or PAU, with the curves suggesting that the high intensity intervention has a greater probability of being the more cost-effective option, irrespective of the willingness to pay for improved effects. For example, at zero willingness to pay for additional true positives identified per practice, there is a 46% probability that the high intensity intervention is the more cost-effective option, compared to 41% for PAU and 13% for the low intensity intervention. This rises to 68% at a willingness to pay of £10,000 (compared to 18% for PAU and 14% for low intensity) and to 77% at a willingness to pay of £20,000 (compared to 10% for PAU and 13% for low intensity).

In terms of the low intensity intervention, despite being found to be more effective and cheaper than PAU, on average, Figure 6 shows that the low intensity intervention has a lower probability of being cost-effective than PAU up to values of willingness to pay of around £15,000 per additional true positive identified per practice. This is due to large and overlapping confidence intervals and the dominance of the high intensity intervention, such that when monetary net benefits for low intensity are higher than for PAU, monetary net benefits for high intensity are commonly higher still. Comparing low intensity to PAU alone, illustrated in Figure 7, shows that the low intensity intervention dominates PAU irrespective of willingness to pay, when the influence of the high intensity intervention is removed.

References

1. Yung AR, Phillips LJ, Yuen HP, McGorry PD. Risk factors for psychosis in an ultra high-risk group: psychopathology and clinical features. *Schizophr Res* 2004;67(2-3):131-142.
2. Yung AR, Phillips LJ, Yuen HP, Francey SM, McFarlane CA, Hallgren M, et al. Psychosis prediction: 12-month follow up of a high-risk ("prodromal") group. *Schizophr Res* 2003;60(1):21-32.
3. Miller TJ, McGlashan TH, Rosen JL, Somjee L, Markovich PJ, Stein K, et al. Prospective diagnosis of the initial prodrome for schizophrenia based on the Structured Interview for Prodromal Syndromes: preliminary evidence of interrater reliability and predictive validity. *Am J Psychiatry* 2002;159(5):863-865.
4. Curtis L. Unit Costs of Health and Social Care 2012. Personal Social Services Research Unit. 2012.
5. Department of Health. National Schedule of Reference Costs 2011-12 for NHS trusts and NHS foundation trusts. [https://wwwgovuk/government/publications/nhs-reference-costs-financial-year-2011-to-2012]. Accessed on 8th May, 2013.
6. Valmaggia LR, McCrone P, Knapp M, Woolley JB, Broome MR, Tabraham P, et al. Economic impact of early intervention in people at high risk of psychosis. *Psychol Med* 2009;39(10):1617-1626.
7. McCrone P, Craig TKJ, Power P, Garety PA. Cost-effectiveness of an early intervention service for people with psychosis. *Br J Psychiatry* 2010; 196(5):377-382.
8. Moscarelli M, Capri S, Neri L. Cost evaluation of chronic schizophrenic patients during the first 3 years after the first contact. *Schizophr Bull* 1991;17(3):421-426.
9. Briggs A, Claxton K, Sculpher M. Decision analytic modelling for the evaluation of health technologies. Oxford University Press, 2006.

10. Fenwick E, Claxton K, Sculpher M. Representing uncertainty: the role of cost-effectiveness acceptability curves. *Health Economics* 2001;10(8):779-787.

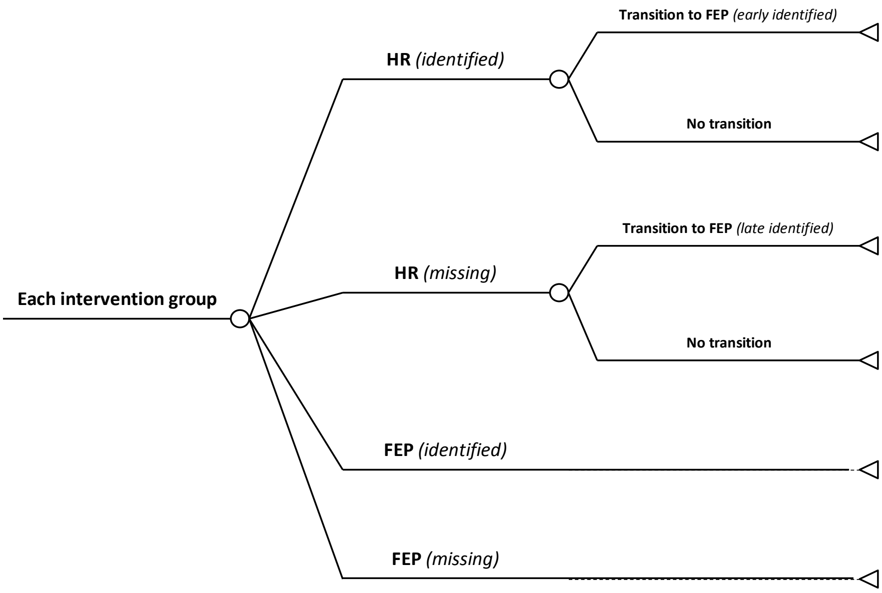

HR=high risk; FEP=first episode psychosis

Figure 1. Model structure

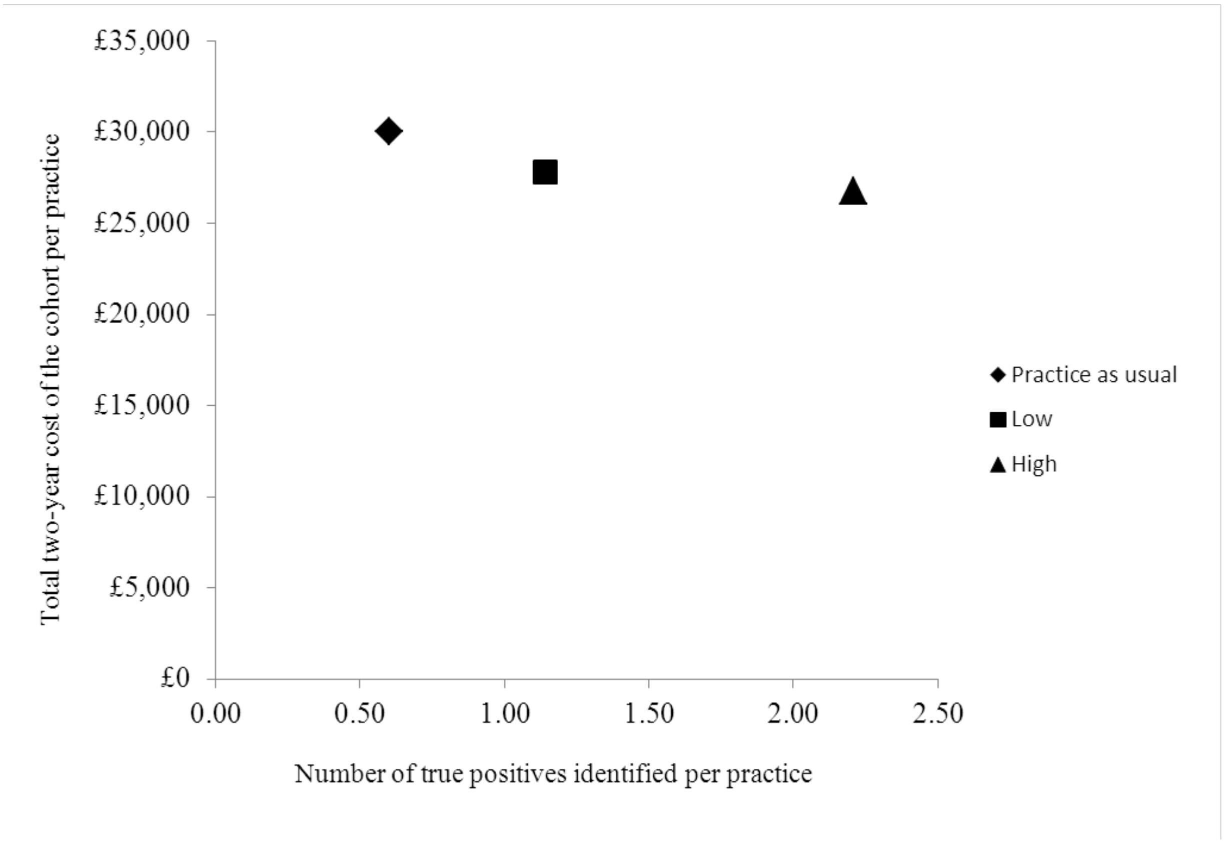

Figure 2. Total costs and effectiveness by intervention group

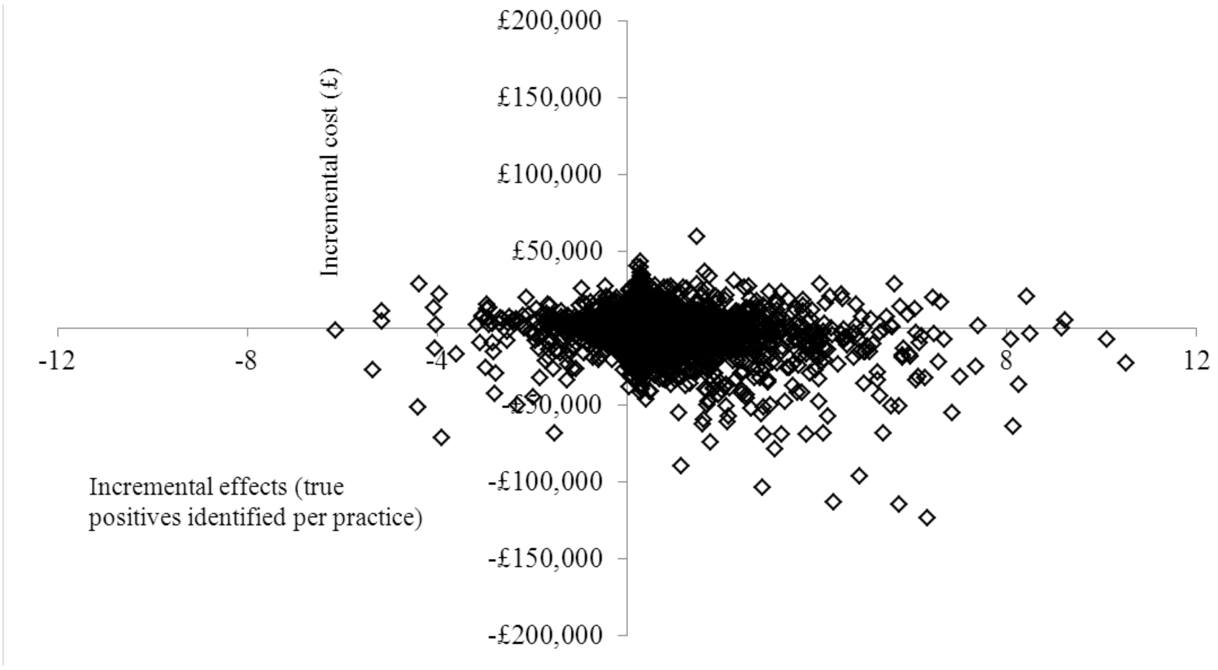

Figure 3. Cost-effectiveness plane for low intensity intervention versus practice as usual

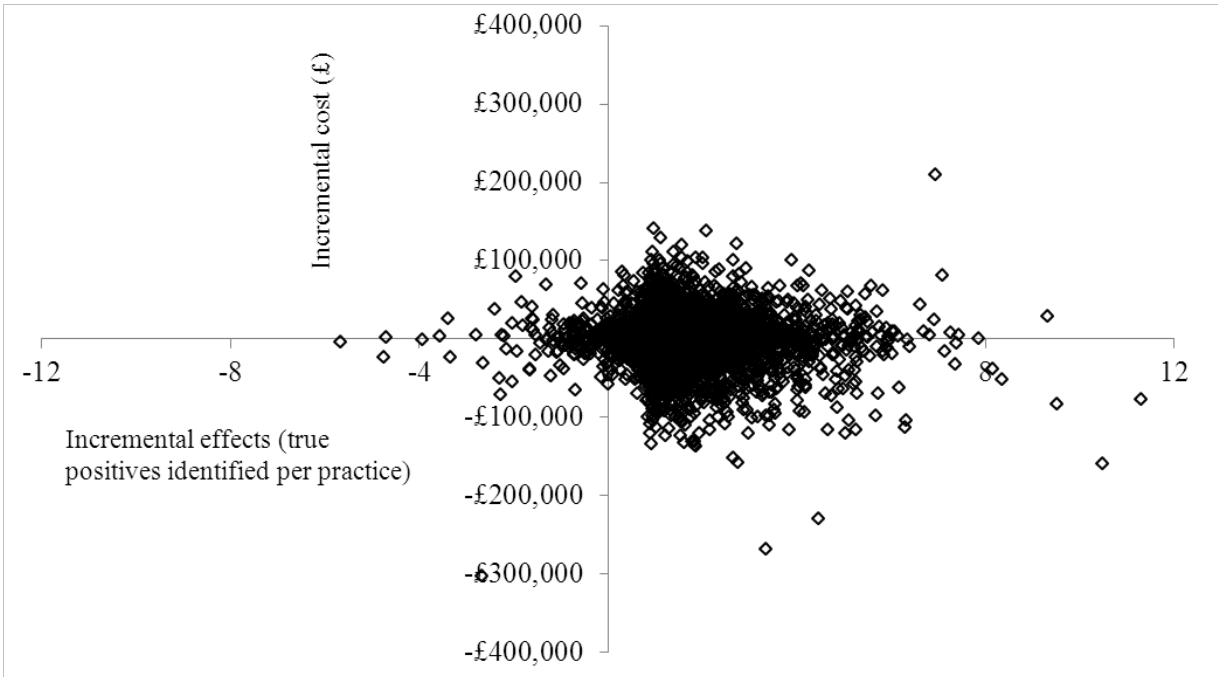

Figure 4. Cost-effectiveness plane for high intensity intervention versus practice as usual

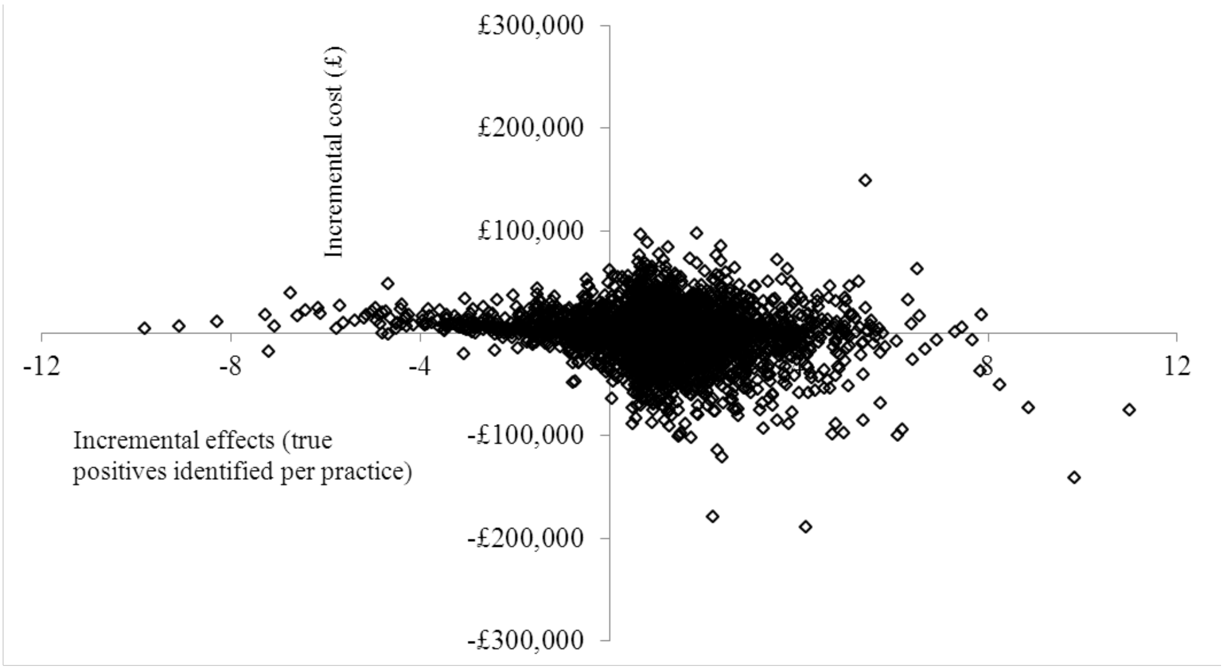

Figure 5. Cost-effectiveness plane for high intensity versus low intensity intervention

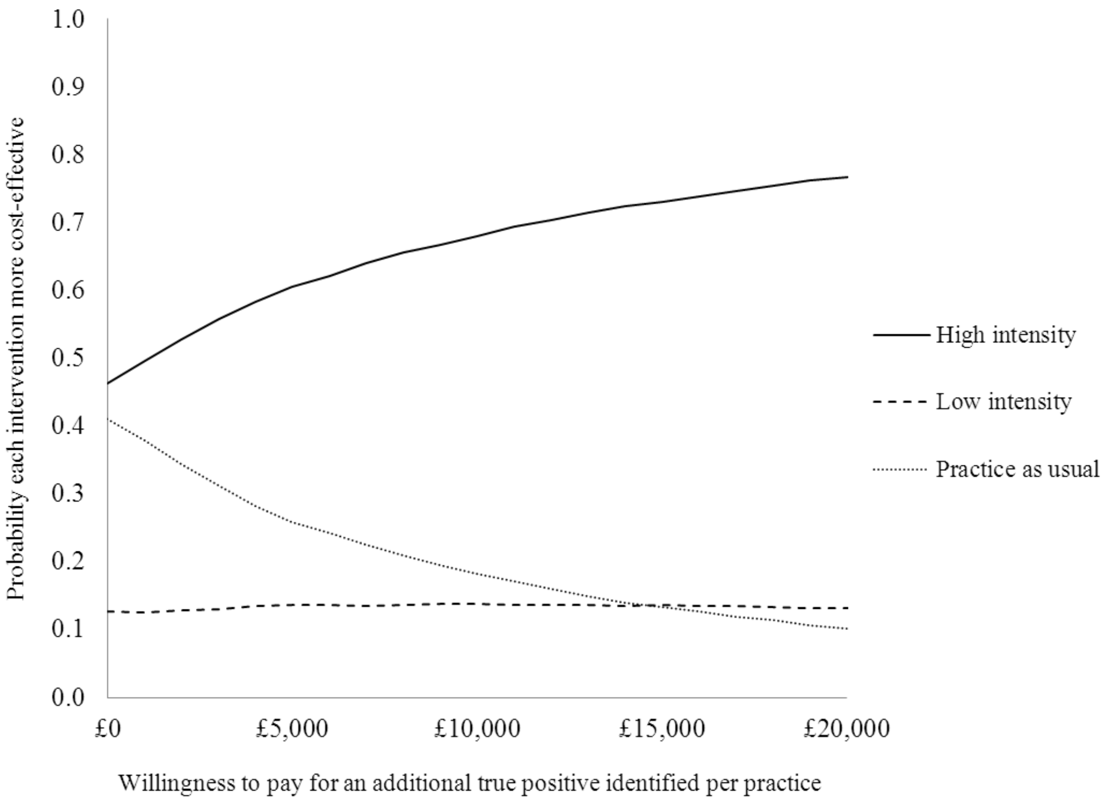

Figure 6. Cost-effectiveness acceptability curves showing probability that each of the three interventions is the more cost-effective

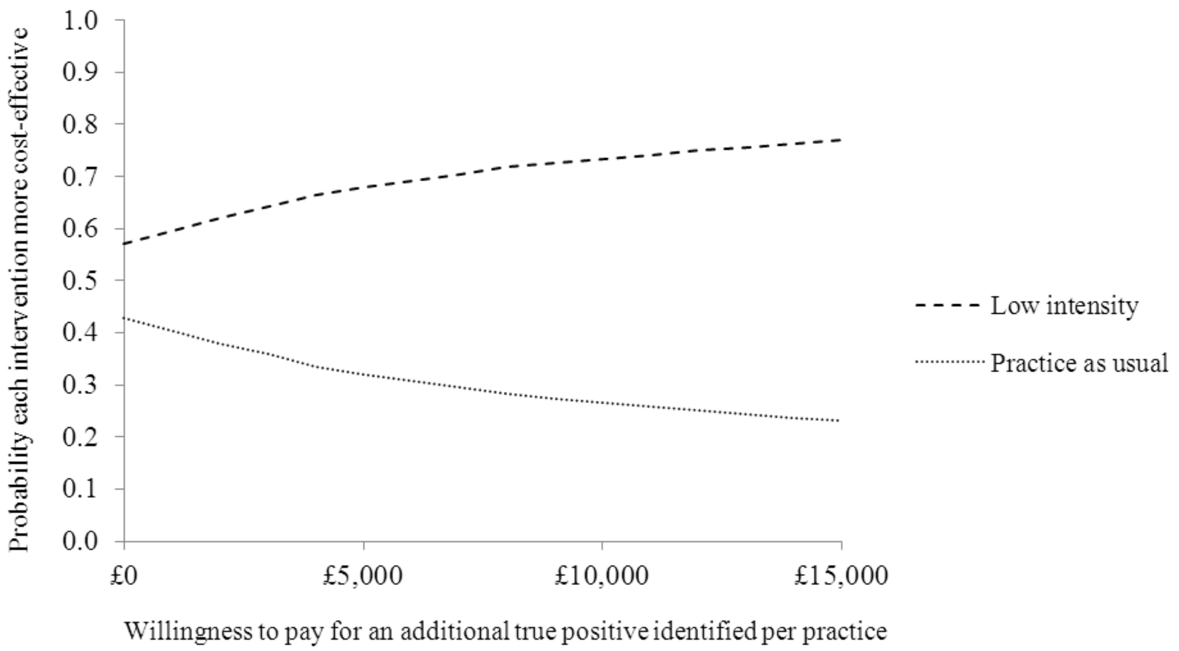

Figure 7. Cost-effectiveness acceptability curve showing probability that the low intensity intervention is more cost-effective than PAU

Table 1. Model input parameters

Parameter	High intensity	Low intensity	Practice as usual	Source
Number of identified HR per practice	0.92	0.46	0.20	Trial data
Number of unidentified HR per practice	0.00	0.46	0.72	Assumption
Number of identified FEP per practice	1.29	0.68	0.40	Trial data
Number of unidentified FEP per practice	0.00	0.61	0.89	Assumption
Transition rates to FEP in identified HR	0.12	0.12	0.12	Trial data
Transition rates to FEP in unidentified HR	0.35	0.35	0.35	Valmaggia et al, 2009[6]
Intervention costs per practice	£1,459	£14.09	£0.00	Trial data
Number of false positives per practice	2.24	0.89	0.78	Trial data
Cost of false positives per practice	£224	£89	£78	Curtis, 2012[4]
Cost of treating identified HR per annum	£1,250	£842	£4,375	Trial data; Curtis, 2012[4]
Cost of treating unidentified HR in year 1	£732	£732	£732	Valmaggia et al, 2009[6]
Cost of treating unidentified HR in year 2	£366	£366	£366	Valmaggia et al, 2009[6]
Cost of treating identified FEP per annum	£8,945	£8,945	£8,945	McCrone et al, 2010[7]
Cost of treating unidentified FEP per annum	£10,351	£10,351	£10,351	McCrone et al, 2010[7]
Cost of treating those identified who transition to FEP	£8,945	£8,945	£8,945	McCrone et al, 2010[7]
Cost of treating those unidentified who transition to FEP	£10,351	£10,351	£10,351	McCrone et al, 2010[7]

HR=high risk; FEP=first episode psychosis

Table 2. Calculation of the cost of the low and high intensity interventions

Item	Total resource use	Unit cost	Total cost
Low intensity group			
Leaflet printing	358	£0.60	£215.52
Leaflet postage	358	£0.50	£179.00
Total cost for 28 practices			£394.52
**Mean cost per low intensity practice**			**£14.09**
High intensity group			
Leaflet printing	294	£0.60	£176.99
Leaflet postage	294	£0.50	£147.00
DVD	n/a	n/a	£3,450.00
Session 1 information pack	173	£3.37	£583.01
Session 1 GP attendance	111 hours	£118/hour[4]	£13,098.00
Session 2 information pack	173	£3.37	£583.01
Session 2 GP attendance	93 hours	£118/hour[4]	£10,974.00
Newsletter printing	882	£0.15	£132.30
Newsletter postage	882	£0.05	£44.10
Liaison practitioners	0.12 of a year	£46,990 per year*	£5,817.79
Total cost for 24 practices			£35,006.20
**Mean cost per high intensity practice**			**£1,458.59**

* Includes employer costs, overheads and indirect time

Table 3. Probabilistic sensitivity analysis parameters

**Interventions**	**Base-line value**	**Distribution**	**Alpha**	**Beta**	**Source**
Number *of identified HR per practice*:					
PAU	0.20	Gamma (SD 0.50)	0.16	1.25	Trial data
Low-intensity	0.46	Gamma (SD 1.00)	0.21	2.17	Trial data
High-intensity	0.92	Gamma (SD 1.01)	0.82	1.12	Trial data
Number of identified FEP per practice:					
PAU	0.40	Gamma (SD 0.61)	0.43	0.93	Trial data
Low-intensity	0.68	Gamma (SD 0.94)	0.52	1.31	Trial data
High-intensity	1.29	Gamma (SD 1.16)	1.23	1.05	Trial data
Number of false positive *per practice*:					
PAU	0.78	Gamma (SD 1.09)	0.52	1.51	Trial data
Low-intensity	0.89	Gamma (SD 1.2)	0.55	1.61	Trial data
High-intensity	2.24	Gamma (SD 2.35)	0.91	2.47	Trial data
Cost of treating identified HR in year 1:					
PAU	£2,613.33	Gamma (SD £5,265.03)	0.25	10,607.24	Trial data
Low-intensity	£233.93	Gamma (SD £377.15)	0.38	608.22	Trial data
High-intensity	£533.25	Gamma (SD £737.80)	0.52	1,020.81	Trial data
Cost of treating identified HR in year 2:					
PAU	£1,762.11	Gamma (SD £3,202.64)	0.30	5,820.66	Trial data
Low-intensity	£607.61	Gamma (SD £1,345.91)	0.20	2,981.27	Trial data
High-intensity	£716.53	Gamma (SD £1,776.54)	0.16	4,404.49	Trial data
Cost of treating identified FEP (18 months):					
Assumed to be the same for all three intervention groups	£13,418.04	Gamma (SD £15,085.99)	0.79	13,345.81	Uplifted from McCrone et al, 2010
Cost of treating unidentified FEP (18 months):					
Assumed to be the same for all three intervention groups	£15,526.79	Gamma (SD £17,401.31)	0.80	15,345.05	Uplifted from McCrone et al, 2010

HR=high risk; FEP=first episode psychosis

Table 4. Proportion of replications from the probabilistic sensitivity analysis falling into each quadrant of the cost-effectiveness plane for each two-way comparison

Intervention under evaluation	Comparison	Southeast quadrant[1]	Northeast quadrant[2]	Southwest quadrant[3]	Northwest quadrant[4]
High intensity	Practice as usual	50.52%	46.54%	1.32%	1.62%
Low intensity	Practice as usual	50.54%	37.26%	6.56%	5.64%
High intensity	Low intensity	43.76%	44.64%	3.16%	8.44%

1 Intervention under evaluation is associated with lower costs and greater effects – intervention under evaluation *dominates* the comparison group
2 Intervention under evaluation is associated with higher costs and greater effects – trade-off between the intervention under evaluation and the comparison group
3 Intervention under evaluation is associated with lower costs and lower effects – trade-off between the intervention under evaluation and the comparison group
4 Intervention under evaluation is associated with higher costs and lower effects – intervention under evaluation is *dominated* by the comparison group

Leaflet delivered to low-intensity and high-intensity practices

Detection of At Risk Mental States for Psychosis (ARMS)

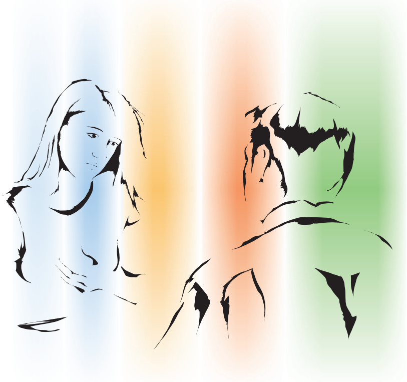

Early detection of psychosis helps

- It halves the risk of suicide
- It improves outcomes
- It can change the course of psychosis

Look out for people at risk of psychosis

- Young people rarely volunteer unusual experiences related to a developing psychotic episode
- They often do complain of losing concentration, feeling 'uneasy', suspiciousness about others, and problems with friends

Ask further questions!

- Ask probing questions about their thoughts, fears, concerns, relationships with other people, performance at school or at work, and recent 'odd experiences'

Do they feel people are watching them or giving them a hard time for no reason?

Do they feel, hear or see things that others cannot?

Do they feel everyday things have a special meaning just for them?

Do they have a feeling that something odd is going on that they cannot explain?

Using the following criteria, refer people aged 16 - 35 to cameo Early Intervention Services

Anybody with *one* of the following:

1x

- Unusual or bizarre thoughts or beliefs, not compatible with their own culture ☐
- Increasing unwarranted suspicion about others ☐
- Hallucinations or inexplicable perceptions of any kind ☐

Or a family history of psychotic illness *plus* one of the following:

1x

- Noticeable deterioration in communication skills ☐
- Thoughts of suicide or uncharacteristic aggressive behaviour ☐
- Deterioration in functioning for at least one month ☐

People with alcohol or substance misuse are not excluded

Cameo Early Intervention Service
Block 7 Ida Darwin, Fulbourn, CB21 5EE
T 01223 884 360 Fax 01223 884 362

Funded by the National Institute for Health Research
Hosted by the Cambridgeshire and Peterborough NHS Foundation Trust
In collaboration with the University of Cambridge
Adopted by the Mental Health and Primary Care Research Networks
